# Enhanced Performance of an Acoustofluidic Device by Integrating Temperature Control

**DOI:** 10.3390/mi15020191

**Published:** 2024-01-27

**Authors:** Mehrnaz Hashemiesfahan, Pierre Gelin, Antonio Maisto, Han Gardeniers, Wim De Malsche

**Affiliations:** 1 µFlow Group, Department of Chemical Engineering, Vrije Universiteit Brussel, 1050 Brussels, Belgium; pierre.gelin@vub.be (P.G.); antonio.maisto@vub.be (A.M.); 2 Mesoscale Chemical Systems Group, MESA+ Institute for Nanotechnology, Faculty of Science and Technology, University of Twente, 7500 AE Enschede, The Netherlands; j.g.e.gardeniers@utwente.nl

**Keywords:** acoustofluidics, temperature control, acoustic streaming

## Abstract

Acoustofluidics is an emerging research field wherein either mixing or (bio)-particle separation is conducted. High-power acoustic streaming can produce more intense and rapid flow patterns, leading to faster and more efficient liquid mixing. However, without cooling, the temperature of the piezoelectric element that is used to supply acoustic power to the fluid could rise above 50% of the Curie point of the piezomaterial, thereby accelerating its aging degradation. In addition, the supply of excessive heat to a liquid may lead to irreproducible streaming effects and gas bubble formation. To control these phenomena, in this paper, we present a feedback temperature control system integrated into an acoustofluidic setup using bulk acoustic waves (BAWs) to elevate mass transfer and manipulation of particles. The system performance was tested by measuring mixing efficiency and determining the average velocity magnitude of acoustic streaming. The results show that the integrated temperature control system keeps the temperature at the set point even at high acoustic powers and improves the reproducibility of the acoustofluidic setup performance when the applied voltage is as high as 200 V.

## 1. Introduction

Acoustofluidics is a research field that integrates acoustics and microfluidics to address challenges and research questions in biology, medicine, chemistry, engineering, and physics [1]. Such a technique manipulates micro- or nanoscale particles in a contactless and biocompatible manner. It has been applied in many fields, such as biological research, chemical analysis, and disease diagnosis [2], and in bioanalytical chemistry to separate specific substances from mixtures [3], separate cells or proteins and DNA, enhance chemical reactions, manipulate cells, and study cell mechanics [2]. 

The working principle of acoustofluidics is as follows: a bulk acoustic standing wave is a type of acoustic wave that propagates through a medium and creates a standing wave pattern. The standing wave pattern creates regions of high and low pressure that can be used to manipulate particles [4]. Standing bulk acoustic waves in a rectangular channel create two effects: liquid streaming generates a drag force on the particles and scattering of acoustic pressure waves in the liquid results in a radiation force along the width of the channel [5]. 

Since the emergence of acoustofluidics, many studies focused on findings that contribute to the ongoing efforts to enhance the performance and applicability of acoustofluidic technologies for various scientific and industry applications. Stringer et al. provide an overview of qualitative and quantitative descriptions of the surface acoustic mechanism and recent developments in this field. This study concludes that there are many ways to enhance the performance of an acoustofluidic setup and choose the appropriate device geometry, electrode design, and various methodologies, depending on the application that it is aimed for [6].

If the end application requires high throughput and sorting performance improvement, changing the microchannel structure can allow for higher flow rates [2]. A previous study on an adaption to channel design describes a method for enhancing acoustofluidic mixing in microfluidic devices using sharp-edge acoustofluidics. The study demonstrates rapid and efficient micromixing through acoustic streaming that is induced by the oscillation of the channel sidewall microstructures. The article highlights the importance of optimizing the designs of the devices to achieve excellent mixing performance and fast mixing speed in less time. Sharp-edge-based acoustofluidic devices hold promise for a wide variety of microfluidic applications, including pumping, particle and cell trapping, and rapid mixing [7]. 

Efforts made to simplify the supportive equipment for acoustofluidic devices also contribute to improving the performance of acoustofluidic setups. This includes, for example, the integration of a programmable acoustofluidic pump to replace syringe pumps and the use of a cell phone, a modified speaker, and a portable microscope for high-demand acoustofluidic pumping and mixing [8]. The choice of substrate material used for fabricating the microfluidic chip and the steps of the fabrication can be optimized to further improve the performance of acoustofluidic setups. For instance, in the case of using bulk standing acoustic waves, one of the best options is using silicon microchips sealed with glass, as fabrication steps can be easily optimized and are straightforward [4].

The effect of microchannel depth on acoustic experiments was studied, and the thickness of a microdevice was found to strongly influence its ability to focus particles by means of acoustic radiation forces. Thinner devices are more affected by mechanical stress from acoustic waves; therefore, compared to thicker devices, they can achieve a higher degree of acoustic focusing. In addition to this, the frequency of acoustic waves is also essential for proper acoustic focusing, but it is restricted by the dimensions of the microchannel. The use of thin glass for closing microfluidic channels allows for more diverse microchannel designs, which may not be advantageous for observing particles in fast flow [9].

In soft robotics, hierarchical nanotexturing of piezoelectric films enables them to function as active microfluidic actuators. Creating nanostructured surfaces that are simultaneously slippery and sticky demonstrates the efficient fluid transportation on surfaces with arbitrary shapes, including inclined, vertical, and inverted three-dimensional surfaces. The unique combination of topographical and chemical cues in hierarchical nanotexturing allows for the manipulation of fluid behavior on complex and flexible surfaces. This research has significant implications for the development of acoustofluidic devices that can operate on nonplanar surfaces [10].

It has been shown that an increased liquid temperature significantly affects the acoustic radiation force and streaming and reduces their effectivity [11]. Therefore, considering the effect of temperature rise when designing and operating acoustofluidic devices is essential.

Acoustic streaming provides a simple and effective way to induce fluid mixing [12]. Higher-power acoustic streaming can produce more intense and rapid flow patterns, leading to faster and more efficient fluid mixing. A study on rapid acoustofluidic mixing via ultrasonic surface-acoustic-wave-induced acoustic streaming demonstrated that the velocity of the acoustic streaming flow is proportional to acoustic power density [13]. On the other hand, evidence shows that higher power can lead to higher temperatures in acoustofluidic chips. In a paper published in 2021, a tracer dye was used to measure the temperature change in a microchannel induced by the thermal effect of standing surface acoustic waves (SSAWs) in microfluidics. The results show that SSAWs can heat the immobile fluid rapidly to 80 °C and that the flow rates can influence the temperature of the liquid [14]. Furthermore, the temperature of the liquid in the microchannel rises as the input voltage increases [15]. The relationship between power and temperature may depend on various factors, such as the chip design, liquid type, and additional cooling mechanisms.

Temperature increase in an acoustofluidic device causes acoustic cavitation in the microchannel. Acoustic cavitation is an evaporation process that arises from pressure reduction below the vapor pressure of the bulk matrix [16]. It has been suggested that cavitation can occur in suspensions saturated with ambient air, even with low-intensity acoustic waves. This hypothesis is supported by previous studies where cavitation occurred at a frequency of 1 MHz at similar voltages [17]. 

A study by Herbert et al. provides valuable insights into the differences and the gap between experimental and predicted data for cavitation pressure in water. They investigated the mechanical tension (negative pressure) that liquid water can sustain before cavitation occurs. Their study focused on the temperature-dependent nature of this quantity. Based on their research, pure water at ambient conditions can sustain enormous negative pressures for a prolonged time before cavitating. The data obtained from the study present a lower envelope for the low-temperature data, which are known to overestimate the cavitation pressure [18]. There is also proven evidence that temperature rise increases cavitation [19], which is another reason to emphasize temperature control systems for acoustofluidic setups. 

The acoustic power generated over time is strictly linked to the actuation power supplied to the piezoelectric element. Without cooling, the temperature can rise above 50% of the Curie point of the piezoelectric material, which defines the upper limit of the working range. Such operative conditions can accelerate the aging degradation of the piezoelectric element, resulting in a reduction in performance. For instance, temperature strongly affects the electromechanical coupling factor determining resonance conditions [20]. Therefore, a temperature control system is required for a high actuation power. The review by Dos-Reis-Delgado et al. [21] gives an overview of temperature control systems for microfluidics using different technologies. Examples of such technologies are integrated microheaters, for which other materials can be used, including thin films of platinum [11,22,23,24]. Despite their efficiency, microheaters have a limited feasible temperature range. Custom-built coolers in diamond plates have been reported for chip cooling [25].

**Figure 1:**
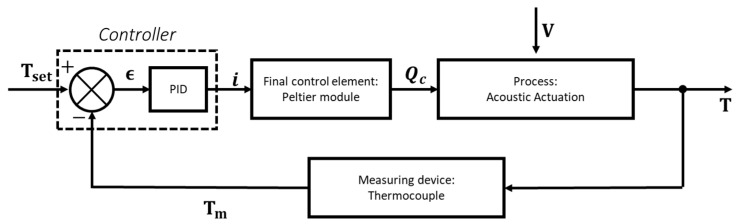
Feedback loop for temperature control.

An alternative to such heaters reported in the literature is the Peltier element to control the temperature [26,27]. The Peltier effect is a thermoelectric phenomenon that results in a temperature gradient between the two sides of a thermoelectric material when a current is supplied. Such an effect makes Peltier elements suitable for heat pumps, transferring the heat generated from the PZT to a heat exchanger and defining the cooling power dependent on current intensity. The cooling/heating cycle is inverted by inverting the current direction inside the element. Peltier elements can be interfaced with microcontrollers to control the cooling power the Peltier generates automatically. Dynamic systems like piezoelectric elements respond to external stimuli, i.e., applied voltage, by generating heat over time. A feedback controller aims to keep the value of a specific output, such as temperature, to a desired value even when the disturbance, i.e., the voltage, is changing randomly. Applied to the control of the temperature of the Piezoelectric, feedback control starts from the measurement of the output temperature. Using a thermocouple, temperature T_m_ is read and compared with a desired temperature from an elaborating unit to define the error. At every cycle, such error ϵ is then elaborated through the characteristic proportional–integral–derivative (PID) functions that define a signal, i.e., current i, to send to the final control element, i.e., the Peltier module. In this way, the cooling power of the Peltier module is modified to accommodate the increase in the temperature generated by the disturbance [28]. A schematic of the feedback control loop of the temperature control system is given in Figure 1.

In this paper, we present a temperature control system integrated into an acoustofluidic setup using bulk acoustic waves (BAWs) to enhance mass transfer and manipulate particles. The system performance was assessed by determining the mixing efficiency and the average velocity magnitude. The results show that the integrated temperature control system keeps the temperature at the set point at high power and improves the acoustofluidic setup performance when the applied potential is as high as 200 (V).

## 2. Materials and Methods

### 2.1. Acoustofluidic Setup with Integrated Temperature Control System

**Figure 2:**
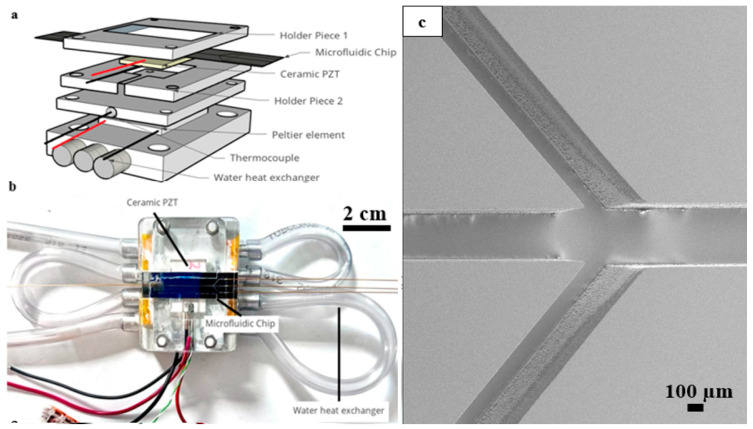
(**a**) Schematic of integrated temperature control system in an acoustofluidic setup (the red and black lines are depicted as positive and negative wire connections); (**b**) a photograph of the tested acoustofluidic setup; (**c**) a scanning electron image of the silicon microchip where three inlets enter the microchannel that is 375 µm wide.

The microfluidic chip, consisting of a microchannel (20 mm × 375 µm × 310 µm) micromachined in a silicon (100) wafer and sealed with a MEMpax^®^ borosilicate glass wafer (both wafers 100 mm diameter), was placed on top of a PZT piezoelement, which is a lead zirconate titanate-based ceramic piezoelement (20 mm × 15 mm × 1 mm, APC International Ltd., Mill Hall, PA, USA) with a thin layer of glycerol in between as a coupling agent. The piezoelement generated a 2 MHz resonance frequency using a frequency generator (AFG1062, Tektronix UK Ltd., Berkshire, UK), and the applied potential was amplified 340 times using an RF power amplifier (N-DP 90, Prana, France) with a power output of 90 Watt. An embedded K-type thermocouple connected to the main PID controller board was used to measure the temperature of the system; based on the temperature difference between the measured temperature and the set point temperature, the controller induced a current from the power supply to the Peltier element, which then cooled down or heated the system. All the setup parts were connected with the help of multiple PMMA holder pieces. Figure 2 depicts the parts and assembly of the setup.

### 2.2. Feedback Control

A proportional–integral–derivative feedback controller (PID) was used to control the temperature of the PZT. In such controllers, the output is given with the equation below:

$$c\left(t\right)=K_{p}\epsilon \left(t\right)+\frac{K_{p}}{\tau_{i}}\int_{0}^{t}\epsilon \left(t\right)dt+K_{p}\tau_{d}\frac{d\epsilon }{dt}+c_{s}\;$$

where $\frac{K_{p}}{\tau_{i}}$ is commonly known as the integral constant; $K_{i}$, $K_{p}\tau_{d}$ are the derivative constants; and $K_{d}$ and $c_{s}$ are the controller bias parameters. Each part of the equation describes the working principle of each function of the controller. The proportional action actuates an output proportional to the error. The higher the gain $K_{p}$ is, the higher will be the sensitivity of the signal to the deviation of the error [28]. The integral action instead causes the controller to repeat the proportional action with a period $\tau_{i}$. Such action is acting until an error is generated, resulting in the removal of small errors [28]. By contrast, the derivative action is capable of predicting the value of the error in the immediate future working as anticipatory control [28]. When dealing with controllers, such constants must be appropriately selected, and they are based on the process of control. A process of tuning is required to properly select the constants. In the case of the temperature control of the experiments, tuning was performed with standard tuning methods. The constants found were $K_{p}=36$, $K_{i}=6$, $K_{d}=4$.

### 2.3. Temperature Evolution

The system temperature change versus time with temperature control was measured. The whole setup and the temperature control system were connected for 120 s, while the frequency generator was kept on at 2 MHz, and an amplified potential of 200 V was applied to the piezoelectric element (indicated with PZT, the material of the element). The change in the system temperature without temperature control was measured in almost the same condition as mentioned before but this time with a turned-off cooling bath and a disconnected Peltier element.

### 2.4. Mixing Efficiency (Index) Experiments

For mixing index experiments, two samples with filtered DI water were prepared: one dyed with florescent (fluorescein sodium salt, Sigma-Aldrich, Taufkirchen, Germany) and the other without the dye. With constant pressure, the dyed water was pumped from the inlets on the two sides into the channel, and the undyed water was pumped from the middle inlet. The mixing of these two samples was carried out at a 2 MHz resonance frequency, and a potential of 30–270 V was applied. As the fluorescence intensity is proportional to the dye concentration, the intensity value can be used as an indication of concentration [29]. The intensity information from the images was subsequently extracted in a region under the influence of acoustic streaming. The mixing indexes were extracted in a region under the influence of acoustic streaming from the pixels’ intensities across a grayscale image cross-section delineating a mixing event, and they were calculated by determining the standard deviation of the pixel intensities in the cross-section with a customized MATLAB (MATLAB R2021a) code. The relative mixing index was calculated by considering the following formula, which deducts the ratio from 1 in percentage form [30]:

$$\eta =1\;–\;\left(\frac{\sqrt{\frac{1}{n}\sum_{i=1}^{n}{\left(I^{\prime }-I_{I,\infty }^{\prime }\right)}^{2}}}{\sqrt{\frac{1}{n}\sum_{i=1}^{n}{\left(I_{I,0}^{\prime }-I_{I,\infty }^{\prime }\right)}^{2}}}\right)$$

### 2.5. Determination of Average Velocity Magnitude of Acoustic Streaming 

Fluorescent monodisperse polystyrene particles of 1 µm in size (Microparticles GmbH, Berlin, Germany), diluted to 0.005 *w*/*v*% in water, were pumped into the channel, and movies were recorded while applying acoustic force to the chip. A set of movies was made at a 2 MHz resonance frequency, and a potential of 30–270 V was applied. The velocity of particles as a function of their position in the channel was studied and processed with a customized MATLAB (MATLAB R2021a) code. The code was used to calculate the velocities by dividing the distances between consecutive positions by the time step. 

## 3. Results and Discussion

The results obtained from this study show that the performance of an acoustofluidic setup, using standing bulk acoustic waves for mass transfer and particle manipulation, can be improved at high power when a temperature control system is integrated. 

**Figure 3:**
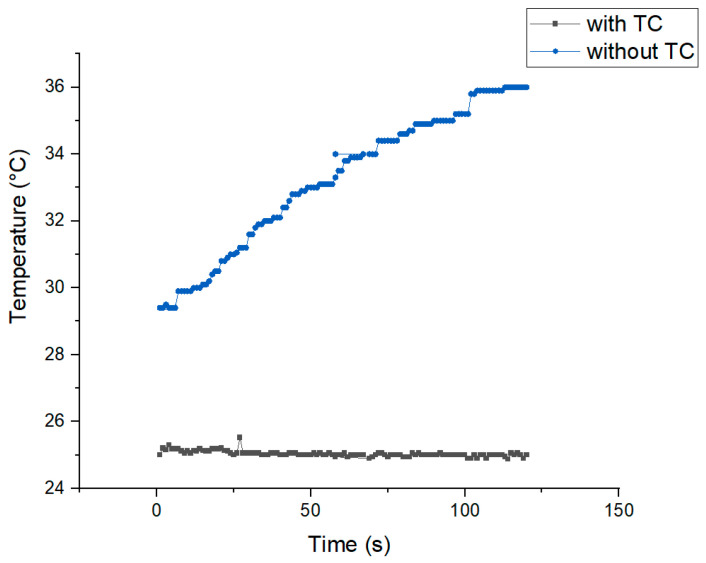
Temperature versus time graph with and without using temperature control (TC) (set point = 25 °C) at 2 MHz resonance frequency and amplified applied potential of 200 V.

In Figure 3, an acoustofluidic setup with and without temperature control is compared. For 120 s, the temperature control system kept the temperature constant at the setpoint temperature, with less than 0.2 °C fluctuations. When the temperature control system was inactive, the temperature increased by roughly 6 °C compared to the setpoint temperature in 2 min. The acoustic properties of liquids, such as the speed of sound and attenuation, are temperature-dependent. In addition, thermal motion and the properties of the particles or cells manipulated using acoustic forces can be affected by the rise in temperature, impacting the experimental outcomes. It would be easier to control these properties by conducting experiments at a constant temperature, leading to more accurate and reproducible results and eliminating the temperature change as a variable.

**Figure 4:**
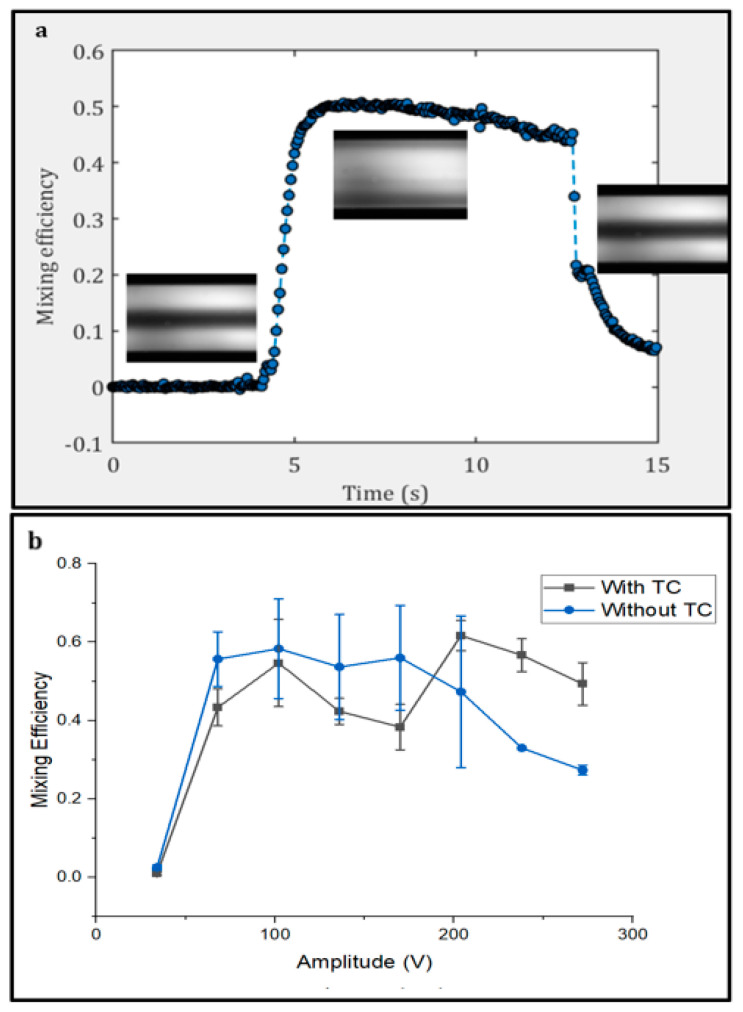
(**a**) Mixing efficiency (index) versus time. The graph displays matching images of distinct stages of experimental steps (first, acoustic setup was off; second, acoustic setup was turned on and mixing occurred; and third, acoustics was turned off); (**b**) mixing efficiency (index) of fluorescent dyed and undyed water in a microchannel measured in different amplitudes while the temperature control was active compared to when it was not (Refer to Videos S3 and S4 in the Supplementary Materials for recorded experimental movies).

Figure 4a shows the final graph that the MATLAB code gives on mixing efficiency index and the experimental photos that match the graph in the different steps. 

**Figure 5:**
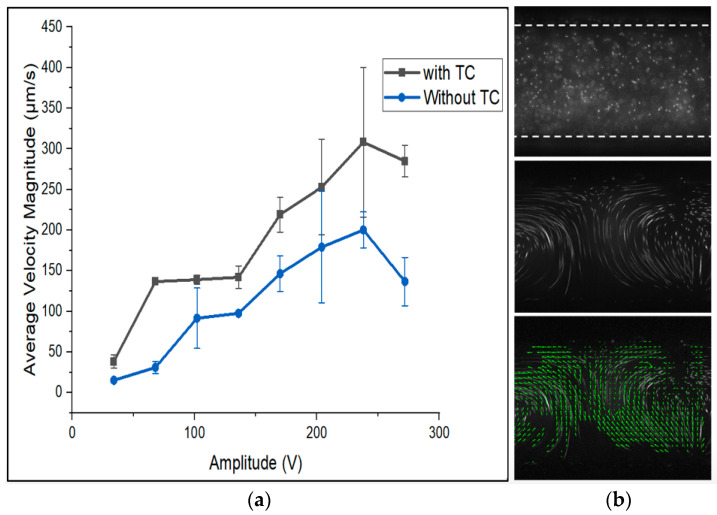
(**a**) The effect of an active temperature control system on the average magnitude of velocity in flow profiles: (**b**) top image: A channel filled with 1 µm polystyrene static particles. Middle image: The flow profiles movement with acoustic energy. Bottom image: The characterized movie shows tiny (green) velocity vectors (Refer to Videos S1 and S2 in the Supplementary Materials for recorded experimental movies).

The results depicted in Figure 4 give a thorough understanding of how the rise in temperature at high potential could affect the mixing performance of a system. Without temperature control, when less than 200 V was applied, the heating of the PZT element caused heating inside the channel, which resulted in a slightly improved mixing efficiency. However, this difference is not significant as the error bars of the two graphs below 200 V considerably overlap and produce a mixing efficiency of a similar magnitude for both conditions. In Figure 5, in which lateral velocity measurements are shown (with smaller error bars), a monotonic behavior is observed. An increase in liquid temperature has several consequences. Firstly, the liquid viscosity changes, leading to changes in flow phenomena such as vortex formation, flow development, and flow patterns in microfluidic channels [31]. In this case, the liquid inside the channel is water and the viscosity of water changes with temperature. Increasing the temperature results in a decrease in the water viscosity [32]. Also, in measuring the mixing index in similar conditions with only differences in the viscosity of liquids, the less viscous the liquid is, the more the mixing index increases, which leads to better mixing performance [33]. This can be explained by the more prolonged compression and expansion cycles of acoustic waves [34]. 

At 200 V, the mixing efficiency improved by 25–30% when the temperature control system was active, and this effect continued to be observed in values beyond 200 V; other factors also played a role (see next paragraph for further discussion). An increase of 1–2 °C in the temperature in a microfluidic device can change the acoustic properties of a system, including the speed of sound. These changes cause a shift in the resonance frequency and, in other cases, based on the microchannel size and the acoustic power, a loss of resonance in the acoustofluidic channel [10].

The results from measuring the mixing index at different amplitudes show a noticeable improvement in the setup performance for values above 200 V. These results indicate that below 200 V, the heating impact was not significant, since the low voltage amplitudes produced less heat [35], and the acoustic setup in this series of experiments was active for less than 1 s. Another approach different from the experimental method Gelin et al. [30] is to obtain information on the mixing efficiency by the use of multiphysics numerical simulation tools. Yin et al. proposed the possibility to evaluate the mixing efficiency by calculating the standard deviation of the concentration in the region interested [36]. By using this approach, it is possible to validate the results.

Indeed, if the setup was active for a longer time, more heat was produced, as shown in Figure 5a. The average velocity magnitude of the flow profiles induced by acoustic streaming showed better performance when the temperature control system was active from the lowest tested potential because the setup was active for 3 s for each experiment.

The velocity trend versus voltage stayed the same with and without the temperature control system, but the condition with an active temperature control system exhibited a higher magnitude of velocity. This graph shows that if the temperature is kept constant, the flow induced by acoustics in the channel has a higher velocity. The speed of sound in liquid changes with temperature, and hence the resonance target frequency is no longer reached under these conditions. The mechanical properties of the chip, such as Young’s modulus, density, and Poisson’s ratio, are temperature-dependent, and they define the dependency of the resonance on temperature [37]. An alternative approach to closed-loop feedback control is possible by evaluating the power generated by the PZT, working in an open-loop regime. It must, however, be pointed out that variations in the temperature during the operation may lead to nonoptimal mixing conditions and eventually the loss of resonance. Additionally, an increase in the temperature can cause boiling inside the microfluidic channels. When the temperature of the liquid reaches its boiling point, a vapor phase emerges, and an increase in pressure causes the liquid to cavitate and form vapor pockets in the channel [38]. To provide some insight, for water in a channel with a hydraulic diameter of 200 µm, an increase in temperature of 2 °C in the walls is enough for the initial formation of vapor bubbles on a heated surface [39]. It is demonstrated that changes in temperature and pressure are often the leading cause of bubble formation in microfluidic setups [40]. Such bubbles may disturb the flow profiles caused by acoustic streaming [41].

Temperature control will play a crucial role in reducing bubble formation and the disturbance inside the channel [42]. Although combining the mentioned temperature control system significantly improves the performance of the acoustofluidic setup, it still has limitations. Figure 4b and Figure 5a both show that the expected increasing trend fails after around 200–240 V applied potential [43]. This shows that although the temperature control system is active after some level of heat production, it cannot cool down the system properly. Figure 2 shows different parts of the setup based on assembly order. As observed from the figure, the thermocouple was used to read the temperature below the PZT. This was carried out to avoid electromagnetic interference and the concomitant disturbance of the temperature readout. As the temperature reached the setpoint in a steady state, the cooling power of the Peltier element reduced in order to keep the surface at the defined temperature. At these conditions, the temperature controller controlled the temperature at the interface between the Peltier element and the PZT. The PZT itself generated heat volumetrically, meaning that the top surface of the PZT was the hotspot of the system. The cooling system seemed to be incapable to account for the increase in the temperature on the top surface of the PZT element at potential values above 200 V. Therefore, when heating produced by the amplified voltage passed this threshold, the system was not able to control the temperature of the system sufficiently. In order to solve this problem, further investigations need to be carried out to find novel ways to measure the chip temperature at the hottest point in the noncontact mode because attaching a thermocouple to the chip is not convenient since it needs proper insulation to avoid electrical interference.

## 4. Conclusions

In this article, we investigated a temperature control system integrated into an acoustofluidic setup using bulk acoustic waves (BAWs) to elevate mass transfer and manipulation of particles. The system performance was tested by mixing efficiency measurements and determining the average velocity magnitude of acoustic streaming. The results show that the integrated temperature control system keeps the temperature at the set point at high power and improves the acoustofluidic setup performance when the applied voltage is as high as 200 V. These results will help enhance various acoustofluidic applications in biomedical research, bioanalytical chemistry, and microfluidics regarding mass transfer enhancement and the manipulation of particles.

## Data Availability

The data presented in this study are available on request from the corresponding author. The data are not publicly available due to data volarization trajectory requirements.

## Supplementary Materials

The following supporting information can be downloaded at: https://www.mdpi.com/article/10.3390/mi15020191/s1, Video S1: Acoustic streaming using 1 µm polystyrene particles with temperature control at 200 V; Video S2: Acoustic streaming using 1 µm polystyrene particles without temperature control at 200 V; Video S3: Mixing of dyed water with temperature control at 200 V; Video S4: Mixing of dyed water without temperature control at 200 V.
